# Multiparametric Remote Investigation in the near-IR through Optical Fiber for In Situ Measurements

**DOI:** 10.3390/s23062911

**Published:** 2023-03-07

**Authors:** Letizia Fede, Gregory Lefrere, Maroun Hjeij, Ronan Le Page, Luiz Poffo, Jean-Marc Goujon, Aymeric Le Gratiet

**Affiliations:** 1 Politecnico di Milano, 20133 Milano, Italy; 2 CNRS, Institut FOTON, Université de Rennes, UMR 6082, F-22305 Lannion, France; 3 CEA-Gramat, F-46500 Gramat, France

**Keywords:** diffuse reflectance spectroscopy, near-IR, non-invasive, tissue, polarization, fiber optics

## Abstract

Diffuse reflectance spectroscopy (DRS) has proven to be a powerful, reliable, and non-invasive optical method for characterizing a specimen. Nevertheless, these methods are based on a rudimentary interpretation of the spectral response and can be irrelevant to understanding 3D structures. In this work, we proposed adding optical modalities into a customized handheld probe head in order to increase the number of parameters in DRS acquired from the light/matter interaction. It consists of (1) placing the sample in a reflectance manual rotation stage to collect spectral backscattered angularly resolved light and (2) illuminating it with two sequential linear polarization orientations. We demonstrate that this innovative approach leads to a compact instrument, capable of performing fast polarization-resolved spectroscopic analysis. Due to the significant amount of data available with this technique in a short time, we observe sensitive quantitative discrimination between two types of biological tissue provided by a raw rabbit leg. We believe that this technique can pave the way for rapid meat quality check or biomedical diagnosis of pathological tissues in situ at an early stage.

## 1. Introduction

Technological assistance for medical diagnosis became an active field of research in the last decade and consisted in investigating tissues and cells in a non-invasive way to enhance the survival rates for cancer patients [1]. Particularly, optical methods proved to be a powerful comprehensive way to fulfill the requirements imposed by the clinical environment. Indeed, such instruments must be user-friendly for all operator profiles, low cost, compact, portable, and efficient when differentiating sensitive tissues.

For decades, numerous spectroscopic works have studied the scattering and absorption properties in in vivo tissues [2,3]. Practically, most of the recent high-performance devices are based on a non-linear process analysis such as Raman scattering or IR absorption [4,5]. Their limitations come from the high costs of the instruments and the special training needed for the operators due to bulky setups that force the operator to manage every aspect of the technique [6]. Among these methods, diffuse reflectance spectroscopy (DRS) or elastic scattering spectroscopy [7,8,9,10] has emerged as a powerful tool offering the possibility of real-time tissue differentiation [11,12]. It is achieved using a broadband white light sent through a fiber into the tissue using inexpensive optical components. After interacting with the tissue, the backscattered light is collected and analyzed using a spectrometer. In such a manner, regions of interest (ROIs) can be deciphered in the reflected spectrum that is highly specific for the absorption and scattering characteristics of the individual tissue properties. Essentially, the heterogeneities inside the sample produce multiple scattering, whereas the absorption is predominantly linked to hemoglobin or water through the tissue [13]. This method has been successfully applied to numerous kinds of tissues, such as skin [14], breast [15], or lung [16] and to molecular concentration monitoring [17]. More particularly, DRS has been mostly beneficial in the medical field toward fast tissue diagnosis in a guided surgery for the near-IR [18], which explains that the most recent advances have allowed for the fast implementation of new solutions such as optimizing optical heads with a polarization modality [19], using multiple coherent light sources simultaneously for diffuse and autofluorescence acquisition in imaging mode [20], and the analysis which is post-processed with machine learning approach [21]. However, analyzing a single spectrum from the reflectance light/matter interaction is not sensitive enough to understand the complex 3D organization of a thick biological sample. On the one hand, the distribution of the light scattering angle erases cumulative factors such as (1) the roughness of the sample surface and (2) the molecular species under illumination [22]. More particularly, the scattering light is overly sensitive to the difference in size or the ratio of the refractive index between scatters and the molecular content at the sub-micrometric scale [23]. On the other hand, the tissue orientation could drastically modify the reflectance spectrum by considering linear polarized light illumination. Certainly, a pointed example could be taken from previous studies that mention the loss of polarization from the randomness induced by pathological tissues [24,25]. On the contrary, highly organized tissues such as muscles exhibit the preferential direction of the reflected light [26].

In this work, we develop a multiparametric DRS setup in the near-IR (NIR) for collecting an optimal set of information from the light backscattering from the tissue surface. More specifically, we have developed an innovative approach based on (1) the acquisition of the polarization-resolved spectra based on the geometrical scattering angle that has never been measured for biological tissues, and (2) an easy-to-handle 3D representation of the variation of the DRS spectra through four distinct physical parameters without requiring any deep knowledge from the operator or computational performances. In this approach, we demonstrate our proficiency in measuring the spectral changes, sensitivity to the molecular content of different tissues in the function of the scattering angle, and the polarization of the light for capturing the geometrical local orientation available from a raw rabbit leg. Here, the near-IR is used in order to opt for a low light dose in the therapeutic window and investigating without damaging the living organisms [27]. Finally, we quantify and correlate spectral scattered measured absolute optical parameters, coming from linear algebraic combinations of orthogonally polarized intensities, such as linear dichroism, through a simple tissue classification method.

## 2. Materials and Methods

### 2.1. Samples Preparation

The scattering sample is a starch granule extracted from raw potatoes. Four potatoes were peeled, shredded into small pieces, and mixed in a container with lukewarm distilled water. The melted liquid was extracted separately from the residual potato pieces through a filter several times. After one hour of decantation, the wet powder was separated from the water and was left to stand for 24 h to obtain a dry, purified starch powder.

The biological specimen of interest for this work was a rabbit leg, known to present strong optical contrast, from the muscle to the tendon regions. The tendon tissue was harvested from a raw rabbit leg purchased from a butcher shop and was cleaned from other connective tissue. The muscular tissue was rinsed several times using an ethanol solution. The thickness of the muscle section is estimated to be around 1 mm down to 0.5 mm. The sections were fixed between a microscope slide and a coverslip, then imaged. In the final application, a raw piece of rabbit leg was directly placed in the sample manual rotation holder.

### 2.2. Experimental Setup and Principle

**Figure 1:**
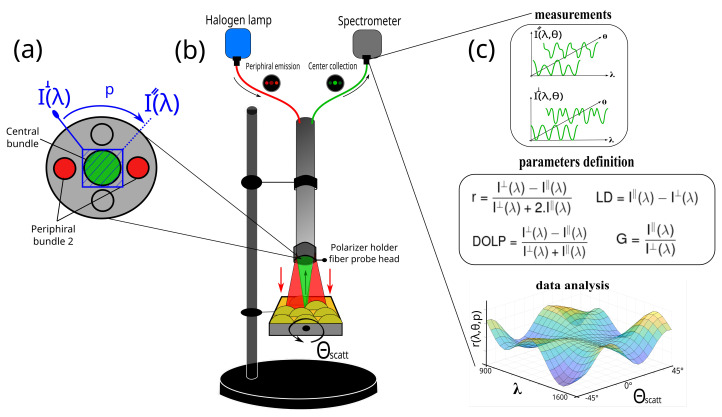
(**a**) Front view schematics of the optical fiber probe head and the polarizer rotation holder. (**b**) Schematics of the experimental in situ setup in DRS architecture. The red-line optical path is emitted from the halogen lamp to the sample and the green-line optical path is the collected backscattered light to the spectrometer. (**c**) Data analysis workflow of the multiparametric approach. $I^{\|}\left(\lambda ,\theta \right)$ and $I^{⊥}\left(\lambda ,\theta \right)$ are the projected collected scattered polarization states; $\theta_{scatt}$ is the scattering angle obtained from the sample placed on a manual rotation holder, with −45° and +45° (step 5°) range; *r*, *LD*, *DOLP*, and *G* are the anisotropy ratio, the linear dichroism, the degree of linear polarization, and the grating factor, respectively.

The single-fiber reflectance system is composed of a 1 W tungsten-halogen white-light source (HL-2000-HP-FHSA, Ocean Optics Inc., Orlando, FL, USA), a portable NIR spectrometer between 900 and 1600 nm (USB2000, Ocean Optics Inc., Orlando, FL, USA) with an integration time of 3.8 ms–10 s, and a single-fiber inox optical probe enclosing fused multimode fibers (designed by FOTON Institute and fabricated by IDIL, Lannion, France) able to both illuminate and collect the light. The head-probe is formed by five optical bundles, containing around 100 active fibers each, and distributed between two anti-symmetrical pairs of distal bundles for emission and a single central bundle for collection as presented Figure 1a.

In this work, only one distal bundle is connected to the lamp and the central bundle sends the light to the spectrometer as presented in Figure 1b. All the devices (light source, spectrometer, and fiber) are connected by SMA connectors that avoid any external interaction with the light being studied.

The fibers have a numerical aperture (NA) of 0.39 allowing for a collection of approximately 80° in scattering angle. In front of the central collection bundle of the probe head, a custom-built rotational mechanical polarization module is placed. It is composed of a linear NIR polarizer film (LP, Sarelec, France) in a manual metallic holder. A stick is attached to the holder to manually rotate the polarizer between the exact 0° and 90° angles. Thus, the collected scattered light is projected into two orthogonal polarization states, giving the spectral intensities noted $I^{\|}\left(\lambda \right)$ and $I^{⊥}\left(\lambda \right)$ as presented Figure 1a. These two measurements are used to determine four parameters as the anisotropy ratio (*r*), the linear dichroism (*LD*), the degree of linear polarization (*DOLP*), and the grating factor (*G*) through the entire spectral range defined as

(1) $$r\left(\lambda \right)=\frac{I^{\|}\left(\lambda \right)-I^{⊥}\left(\lambda \right)}{I^{\|}\left(\lambda \right)+2I^{⊥}\left(\lambda \right)}$$

(2) $$LD\left(\lambda \right)=I^{\|}\left(\lambda \right)-I^{⊥}\left(\lambda \right)$$

(3) $$DOLP\left(\lambda \right)=\frac{I^{\|}\left(\lambda \right)-I^{⊥}\left(\lambda \right)}{I^{\|}\left(\lambda \right)+I^{⊥}\left(\lambda \right)}$$

(4) $$G\left(\lambda \right)=\frac{I^{\|}\left(\lambda \right)}{I^{⊥}\left(\lambda \right)}$$

*r* (−1≤r≤1) is widely used as an indicator of molecular size or diffusion in fluorescence spectroscopy and expresses the preferential excitation of molecules with transition dipoles oriented along a particular polarization direction [28]. *LD* (−1≤LD≤1) corresponds to the difference of two absorption spectra between two orthogonal linear polarizations and is linked to the macroscopic averaged orientation of the molecules [29]. *DOLP* (−1≤DOLP≤1) provides information on the tissue’s ability to reflect linear polarization. The grating factor G (G≥0) is an instrumental preference of the emission optics for $I^{\|}\left(\lambda \right)$ rather than $I^{⊥}\left(\lambda \right)$.

Before recording the sample reflectance $R_{s}\left(\lambda \right)$, any spectrum is flatted by a post-calibration step consisting in measuring the reference from a spectralon (Labsphere) and the background spectra, $R_{ref}$ and $R_{bg}$, respectively, as described in Ref. [30]. Thus, the corrected spectrum from the sample R(λ) is obtained from the formula

(5) $$R\left(\lambda \right)=\frac{R_{s}\left(\lambda \right)-R_{bg}\left(\lambda \right)}{R_{ref}\left(\lambda \right)-R_{bg}\left(\lambda \right)}$$

Finally, the sample is placed in a manual rotation sample holder and at an optimal distance from the fiber head for maximizing the illumination and collection. In such a manner, the manual collection of the scattering light is measured from −45° to +45°, by steps of 5°, according to the fiber optical axis as presented in Figure 1b and introduced an incertitude of the angle estimation of 0.5°, which is acceptable considering that this work is dedicated to measuring comparative parameters. The overall acquisition time by scattering angle is a few seconds, which corresponds to whole measurements in around two minutes. We believe that this time could be reduced by (1) placing the sample in a motorized and automated angular holder and (2) motorizing the two orthogonal polarization orientations in the probe head.

### 2.3. Polarized States Incertitude

For evaluating the accuracy of the polarized light backscattered collection through the fiber, we place a linear polarizer on a spectralon surface after the fiber. Thus, we estimate the incertitude compared to the Malus law by rotating the linear polarizer in the fiber holder. As a result, the inaccuracy of the polarization light collection was estimated to be less than 10% and erased from the divergence of the white source, the spectral response of the LP, and the optical misalignment of the custom-built polarizer holder.

### 2.4. Image Acquisition

The images are collected through a modified commercial Leica DM4000M microscope (Leica Microsystems, Wetzlar, Germany) dedicated to wide-field phase contrast and polarization-resolved microscope. The imaging device is a Sony Tri-CCD 736 × 574 pixels camera (Sony Group Corporation, Tokyo, Japan), and the images are captured and stored using Sarfusoft software (Nanolane, Le Mans, France).

### 2.5. Data Analysis

All the spectra are recorded following the block diagram Figure 1c and processed via Matlab (The MathWorksTM Inc., Natick, MA, USA). Briefly, for both polarization states, spectra of the tissue reflectance are collected for all the scattering angles and the results are presented in a 3D plot, where the axis corresponds to the wavelength (x-axis), the spectral amplitude (z-axis), and the scattering angle (y-axis). Then, the two orthogonal representations are compared to provide the desired parameter mentioned in Section 2.1.

## 3. Results and Dicussions

### 3.1. Starch Granules

The characterization of starch granules under stress or thermal conditions is an emerging research topic for investigating food quality [31]. In order to optimize in vivo and in situ measurements in a robust way, a large number of optical methods have been proposed using polarimetry [32] or non-linear microscopy [33]. Indeed, the chiral supra-molecular conformation of starch exhibits a strong anisotropic emission of the light at a non-incident scattering angle due to its dipoles high-ordered arrangement and offers a potentially powerful dataset using the multiparametric approach described in this work [34].

**Figure 2:**
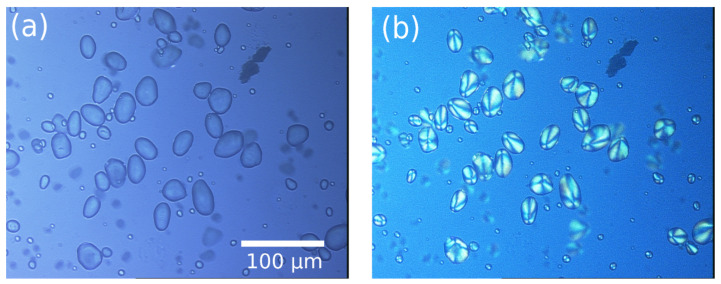
732 × 546 pixel images of starch granules from potato using a Leica microscope customized with tri-CCD Sony camera and Sarfus software. (**a**) White lamp wide-field image and (**b**) crossed polarization-resolved image.

The extracted starch granules are used in this work to illustrate the capability of our technique to measure the polarization optical response of molecules in a turbid medium. The sample is presented under normal and polarization-resolved white source illumination, i.e., crossed polarization, as seen in Figure 2.

As expected, the granules under polarized light in Figure 2b present a well-known imaging quadratic pattern induced by the relative phase difference inside the 3D shell structures depending on the scattering angle. Thus, the scattering beams interfere destructively in the forward direction and constructively out-axis.

**Figure 3:**
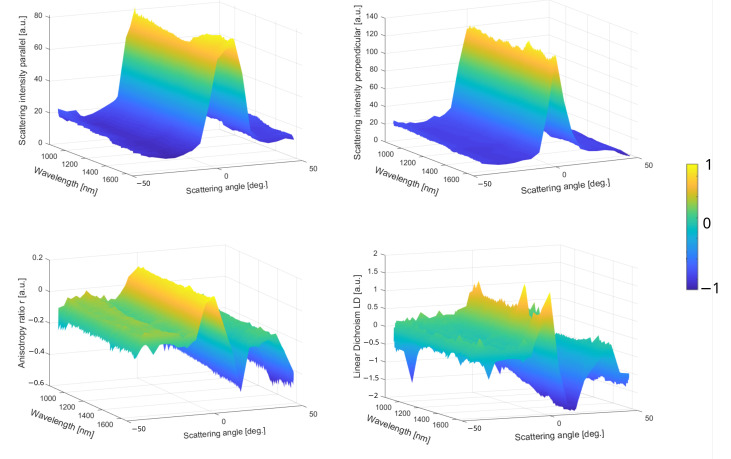
Multiparametric scattering sensing of starch granules from potatoes. (**Top row**) Raw parallel and perpendicular spectra in function of the scattering angle. (**Bottom row**) The *r* and *LD* spectra in function of the scattering angle.

As a next step, the starch granules are placed under white source illumination through the optical probe-head providing the polarization spectra obtained from the calibration procedure detailed using Equation (5), leading to the 3D representation reported in Figure 3.

The raw data are reported in the top row of Figure 3 corresponding to the parallel and perpendicular spectra recorded in the function of the scattering angle provided by the optical setup reported in the Section 2. The sample is placed on a glass microscope slice; thus, no geometrical feature could contribute to any fluctuations in the scattering amplitude. In this case, the light emission is specular, i.e., forward light emission, which is confirmed by the Gaussian curves for both orthogonal polarization states in Figure 3 top. However, the magnitude difference between these two orthogonal scattering spectra, a factor of about 1.5, indicates a privileged light emission orientation as can be visualized in the images in Figure 2. This effect is induced by a preferential dipole transition excitation direction from the illuminated molecules, resulting in anisotropic off-axis polarization emission. Thereby, *r* and *LD* are shown in the bottom row of Figure 3 proving that the sign and magnitude are angular-dependent on the sample orientation since these two parameters are non-null. The *LD* scattering pattern for chiral molecules exhibits two antisymmetric amplitudes related to forward emission, and is consistent with the Kramers–Kronig dispersion relation [35]. The *r* function is almost negative and decreases drastically at a high scattering angle, caused by the specular reflection on the plane surface. The *LD* function is symmetrical with two identical absolute magnitudes in the positive and negative signs. Usually, this pattern is well-observed for the visible wavelength range and *LD* becomes negligible in the NIR. However, the curve is still Kramers–Kronig-consistent in the function of the angle due to the conservation of the averaged macroscopic light emission that implies no change in the complex dielectric function. In order to simplify, *DOLP* and *G* are not presented for this sample, are assumed to be almost inconsequential for such molecules, and exhibit a preferential circular polarized light [36]. In summary, Figure 3 plots express two distinct physical effects from the same illumination dominated by (1) the Mie scattering in Figure 3 top and (2) the polarized light in Figure 3 bottom. More precisely, the top Figure 3 plots indicate the averaged size of the scatters, and comparing both parallel and perpendicular spectral intensities is interesting to obtain a general idea on how the average dipolar orientation is. On the contrary, the bottom Figure 3 plots provide a deeper understanding of the physical or mechanical properties of a material in different spatial directions. Thus, Figure 3 bottom plots could allow for the localization of the chiral molecules hidden in a mixed/bulky environment in a label-free way or give access to different structural order and orientation.

### 3.2. Rabbit Leg

In this work, the application of the optical technique reported in the Section 2 is dedicated to the differentiation of tissues, which are assumed to differ according to (1) the molecular species under illumination, (2) the 3D arrangement, and (3) the averaged orientation.

**Figure 4:**
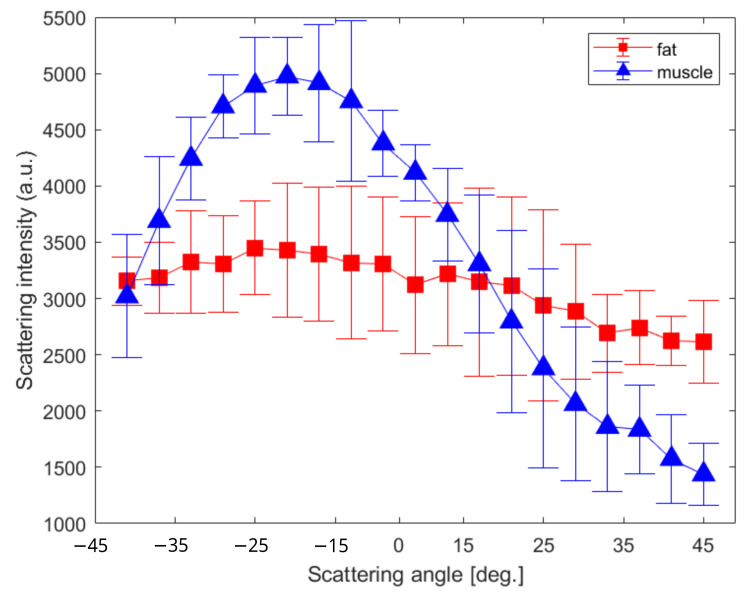
(**a**) Raw rabbit leg with the ROIs presented for the connective/muscular (red) and single muscular–tendon (blue) tissues. (**b**) Total polarized-resolved scattering intensity collected and averaged over each region.

The raw rabbit leg used in this study is shown in Figure 4a in which the two areas of interest are delimited by red and blue squares, corresponding to a mix of connective/muscular features (associated with fat and epithelial cells) and single muscular tissue (tendon) ROIs, respectively.

To correlate the optical fingerprint through the different parameters provided by this instrument with the tissue type under illumination, we selected three ROIs for each area. Figure 4b shows the scatter amplitudes as a function of angle, integrated over the entire spectrum and averaged across the three ROIs for the connective/muscle and tendon areas.

In the blue plot, we can see that the single muscular tissues exhibit a stronger specular behavior compared to the connective/muscular tissues. This could be explained by the Mie theory which mentions an emission diagram linked to the type with a relative refractive index and the size of the scatters [37]. Thus, the unique molecular specie under illumination induces a straightforward reflection with weaker magnitudes at higher scattering angles. Conversely in the red plot, a mixture of different types of scatters that formed the mixed type of tissue (composed of muscle, fat, and epithelial), coupled with a multi-scattering process, produce a stochastic light reflectance indifferently at any scattering angles [38].

**Figure 5:**
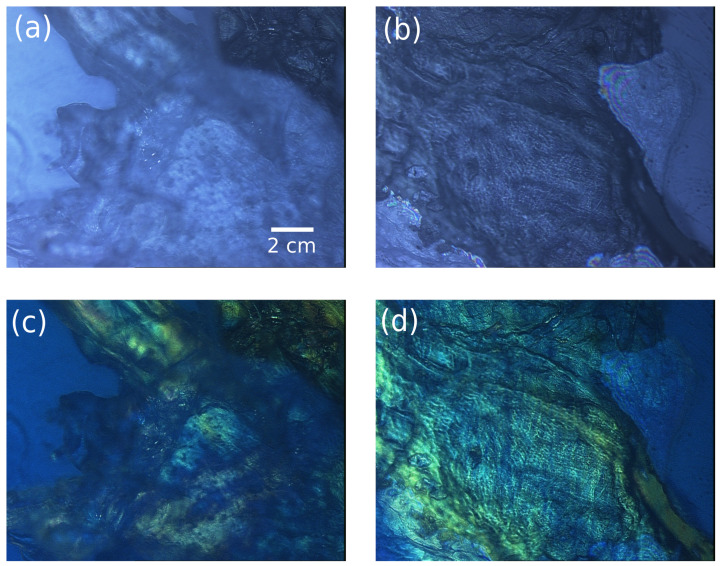
736 × 574 pixel images of a rabbit leg. Images show (**a**,**c**) the connective/muscular tissues and (**b**,**d**) the tendon region (**b**,**d**). The images are obtained under total light intensity (**a**,**b**) and polarized light (**c**,**d**) from a Leica microscope.

More precisely, two typical areas of the specimen have been selected for imaging under widefield microscopy without and with polarized light, as shown in Figure 5.

Figure 5a,c is related to the connective/muscular region and Figure 5b,d corresponds to the tendon of the leg. The colors observed in the polarization images of Figure 5b,d are induced by the dephasing effect of thick and scattered tissues on the polarized light, highlighting specific tissue or biological arrangement. This modality is a powerful tool for optical microscopy since it provides label-free contrasts of the sample organization and drastically improves the quantitative information available from the light–matter interaction [39]. In the images reported in Figure 5, the mixed tissue region (fat, epithelium, and muscle) in Figure 5a,c appears disorganized in all 3D volumes, which explains the blurred area in the images and their bulky aspect. On the contrary, the tendon region in Figure 5b,d appears more organized and well-structured corresponding to the actin–myosin complex that forms the retractable muscle.

**Figure 6:**
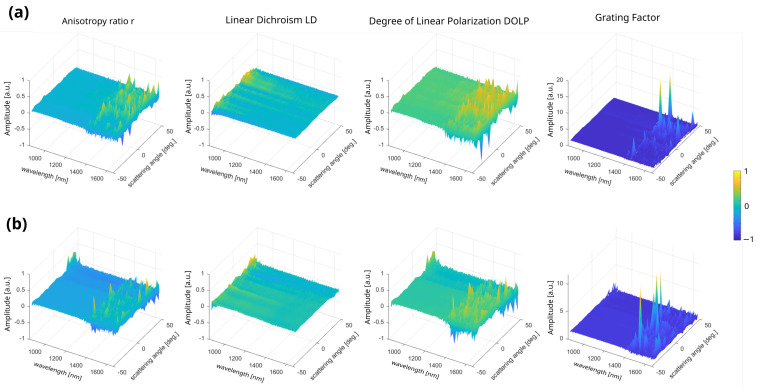
Multidimensional representation of the four averaged parameters studied in this work (**a**) over the connective/muscular ROIs and (**b**) over the tendon ROIs. The parameters *r*, *LD*, *DOLP*, and *G* correspond to the anisotropy ratio, the linear dichroism, the degree of linear polarization, and the grating factor, respectively.

As for the previous starch sample, we propose in Figure 6 the 3D multiparametric representation of both regions visualized in the previous widefield microscopy images, by means of the connective/muscular (top row) and tendon (bottom row) area, averaged overall the ROIs. In this work, three regions of each tissue type were studied and presented enough statistical data to demonstrate the potentiality of the technique to separate the optical response of the two tissues as discussed below.

Comparing the four parameters presented in the Section 2, similarities are observed for *r* and *DOLP*, but they differ between both regions. The reason for this lies in the mathematical definition of these two parameters, which only differ by a factor of two in the intensity ratio [40]. *LD* is becoming a zero parameter for both tissues but exhibits differences at higher scattering angles. Indeed, since *LD* is sensitive to averaged molecular order, at this NIR wavelength range, the two bulky tissues appear to be too similar, and cannot produce any significant dichroism difference in the forward direction. The *r* and *G* parameters exhibit the highest difference spelled out by their extreme sensitivity to the macroscopic biological organization. Since incoherent light interacts more efficiently with weak-ordered structures such as raw thick tissues, it provides enough quantitative information for differentiating both tissues [22].

**Figure 7:**
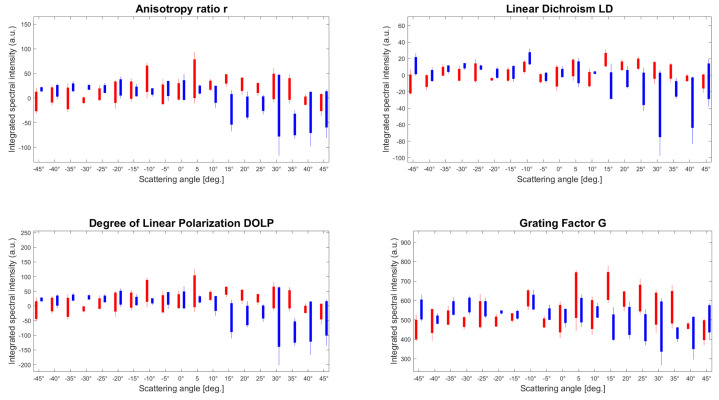
Multidimensional estimation of the four parameters integrated over the whole spectra with the three ROIs per tissue type. The parameters *r*, *LD*, *DOLP*, and *G* correspond to the anisotropy ratio, the linear dichroism, the degree of linear polarization, and the grating factor, respectively. The three spectral integrated values have been reported in a single bar where the red and blue correspond to the connective/muscular and tendon tissues areas, respectively.

To look more closely at the statistical dispersion of the parameter amplitudes and understand the influence of the 3D illumination location, Figure 7 reports the integrated spectra in the function of the scattering angle over all of the three ROIs for the connective/muscular tissues (red) and tendon (blue). For clarity reasons, each bar corresponds to the three integrated spectral values by area related to the scattering angle. In such a manner, the contribution of the negligible magnitudes observed in Figure 6 at lower wavelengths can be silenced. Additionally, such statistical analysis improves the sensitivity of the multiparametric approach and quantifies the degree of randomness of the macroscopic biological organization.

The statistical distribution of the averaged magnitudes of the four parameters from Figure 7 is reported in Table 1. It resumes the mean values and the statistical distribution for the overall scattering angular range which provides a relative estimation of the four polarimetric parameters to discriminate two types of tissue.

From Table 1, the mean distribution for each scattering angle presents a simple marker for discriminating both tissues and the associated standard deviation could be used to evaluate the degree of organization of the specimen. In principle, the surface roughness and molecular content are different depending on the position of the illumination, leading to diverse optical responses on the surface. Table 1 witnesses this diversity of the biological organization through the dispersion of the statistical values. More precisely, the mean and standard deviation for the connective/muscular ROIs are globally not influenced by the incident angle and the measurements are reproducible independently of the tissue location. However, the tendons’ ROIs exhibit a stronger dependence on the light excitation, and the magnitudes of each parameter are clearly more important at high scattering angles compared to the muscle area. For instance, the corresponding standard deviations are twice as high as for the muscle ROIs, except for G. This is caused by the illumination volume and the size of the scatters being different for both tissues. As we mentioned in Figure 4, the connective tissues present a Lambertian-type reflectance, i.e., angularly isotropic, so all the tissues could have similar behavior independently of light direction. In addition, the specificity of each molecule, composed of bulky tissues and numerous different types of molecules, is blurred in the fingerprint averaged by the incoherent illumination. On the contrary, the tendons are thicker and are composed of fewer different molecular species. Therefore, this induces a specular reflection, i.e., directive, and thus, more sensitive to the overall geometry and tissue orientation. This explains the higher standard deviation at a high scattering angle, especially verified by *LD* and *DOLP* in which the discrimination is greater. This is important because any weak modification in the light amplitude would be hidden in a small signal-to-noise ratio (SNR) for a wide scattering angle range. Thus, we can conclude that for such measurement, the most optimal contrast between different biological tissues may be obtained from the collected light at more than 45° related to the illumination direction.

## 4. Conclusions

In this work, we have implemented a multiparametric approach for characterizing raw biological specimens in a remote in situ mode. The instrument prototype is based on a collection of spectra through a customized optical fiber, providing direct, easily handled, and low-cost measurements. We have proposed to measure the optical fingerprint of well-known anisotropic chiral molecules placed on a microscope slide. This preparation suffers from inhibiting the 3D organization contributions but offers the advantage of presenting a dataset in adequation with the literature. In parallel, we have investigated the optical fingerprint of a raw rabbit leg for quantifying the difference between two types of tissues in the angular and polarization-resolved process. In summary, we showed that the Lambertian-type reflection from the connective/muscular tissues is produced by multiple scattering with numerous different molecular species, which can be considered as a bulk region. Thus, this is expressed through small changes of the associated four parameters over the scattering angles. On the contrary, tendon tissues are composed of fewer different species and strongly oriented fibers; thus, the specular reflection expresses a higher angular variation of the multiparametric approach. Furthermore, having access to all these parameters simultaneously allowed for a simpler analysis of how the illuminated sample is organized and discriminating different tissues in situ without preparation of the sample. In future work, we propose an upgrade of the technique to speed up the measurement and simplify the multimodal use of the instrument. First, the collected linear polarizer will be placed in a small motorized rotation stage, synchronized with the acquisition of the spectra by polarization states. In parallel, the sample holder could be synchronized with a stepper motor driver. Second, we propose automating the spectra acquisition without moving parts for measuring clinical tissue to put forward a scoring approach and classification in medical diagnosis.

## Figures and Tables

**Table 1:** Statistical estimation of the averages and the standard deviations of the four polarimetric parameters for each scattering angles from Figure 7. The values correspond to the integrated spectra per scattering angle. *r*, *LD*, *DOLP*, and *G* are the anisotropy ratio, linear dichroism, degree of linear polarization, and grating factor, respectively.

	*r*	*LD*	*DOLP*	*G*
Muscle	14.3 ± 10.6	1.9 ± 3.6	16.8 ± 14.8	536.2 ± 37.6
Tendon	2.4 ± 22.9	−2.7 ± 14.6	−1.4 ± 37.0	516.6 ± 40.4

## Data Availability

The datasets generated and/or analyzed and the Matlab algorithm in the current paper are available from the corresponding author upon reasonable request.
